# Predictive Value of Gray-Matter–White-Matter Ratio on Brain Computed Tomography for Delayed Encephalopathy after Acute Carbon Monoxide Poisoning: A Retrospective Cohort Study

**DOI:** 10.1155/2021/5511290

**Published:** 2021-05-31

**Authors:** Shu Li Wang, Meng Mei Ma, Guang Wei Lv, Meng Zhang, Yu Sen Du, Su Li Zhang, Shun Yi Feng, Yong Li, Yuan Yuan Zhang

**Affiliations:** ^1^Emergency Department, Cangzhou Central Hospital, No. 16 Xinhua Road, Yunhe Qu, Cangzhou City 061000, China; ^2^Graduate School, Tianjin Medical University, Tianjin 300070, China; ^3^Nursing Department, Cangzhou Infectious Diseases Hospital, No. 68 Guangrong Road, Yunhe Qu, Cangzhou City 061000, China

## Abstract

**Background:**

This study is aimed at determining the predictive value of the gray-matter–white-matter ratio (GWR) on brain computed tomography for delayed encephalopathy after acute carbon monoxide (CO) poisoning (DEACMP).

**Methods:**

This retrospective cohort study reviewed 352 patients with acute CO poisoning and who underwent the brain computed tomography test. These patients were admitted to Cangzhou Central Hospital from May 2010 to May 2020. The patients were divided into the DEACMP (*n* = 16) and non-DEACMP (*n* = 336) groups. Pearson's correlation coefficients were computed for correlation analysis. The predictive value of GWR for DEACMP was evaluated by using logistic regression analysis and receiver operator characteristic curves.

**Results:**

The morbidity of DEACMP was 4.5% (16/352). The GWR-basal ganglia, GWR-cerebrum, and GWR-average in the DEACMP group were lower than those in the non-DEACMP group. Correlation analysis indicated that GWR-basal ganglia (*r* = 0.276; *P* < 0.001), GWR-cerebrum (*r* = 0.163; *P* = 0.002), and GWR-average (*r* = 0.200; *P* < 0.001) were correlated with DEACMP. Multivariate logistic regression analysis revealed that reduced GWR-basal ganglia, GWR-cerebrum, and GWR-average were independent risk factors (*P* < 0.001; *P* = 0.008; *P* = 0.001; respectively). Compared with GWR-cerebrum and GWR-average, GWR-basal ganglia had a higher area under the curve of 0.881 (95% confidence interval: 0.783–0.983) with sensitivity and specificity of 93.8% and 68.7%, respectively. The cut-off value of GWR-basal ganglia was 1.055.

**Conclusion:**

GWR, especially GWR-basal ganglia, is an early useful predictor for DEACMP.

## 1. Background

Carbon monoxide (CO) is a colorless, odorless, and nonirritant gas produced by the incomplete combustion of carbon-based compounds [1]. CO poisoning occurs through the inhalation of a relatively high concentration of CO gas due to routine domestic, occupational, and recreational activities and in the wake of large-scale disasters, such as wildfires, floods, and storms. The clinical manifestations of CO poisoning vary from mild symptoms, such as headaches and dizziness, to increasingly severe issues, such as unconsciousness and death. Delayed encephalopathy after acute CO poisoning (DEACMP) is characterized by a series of neurological and psychiatric symptoms, such as movement disorders, cognitive impairment, or affective disorders after several days or even weeks of intermittent periods (pseudorecovery period), along with normal performance in patients who experienced CO poisoning and regained consciousness [2–4].

The worldwide cumulative incidence and mortality of CO poisoning in 2017 were approximately 137 cases and 4.6 deaths per million, respectively [5]. The exact incidence, mortality, and disability rates of DEACMP are unknown given the absence of diagnostic criteria and effective treatment but may fall in the ranges of 3%–40%, 1%–3%, and 20%–25%, respectively [2, 6, 7]. Thus, researchers and clinicians must explore reliable methods for predicting the likelihood of DEACMP development after acute CO poisoning.

Gray-matter–white-matter ratio (GWR) on brain computed tomography (CT) and the ratio of gray matter attenuation to white matter attenuation can be used to predict poor neurological outcomes in patients with hypoxic-ischemic encephalopathy, especially those who suffered from cardiac arrest, extra-axial hematoma, and acute stroke [8–11]. Choi et al. [12] reported that the most common finding was low density in the cerebral white matter, and the second characteristic feature was low density in both globus pallidi. Furthermore, Miura et al. [13] showed that the most common finding was symmetric and diffuse low-density cerebral white matter, which was more advanced in the centrum semiovale and varied in degree from slight to severe. Some information on the change of brain tissue density is available. However, a knowledge gap exists in the predictive value of GWR for DEACMP and needs to be comprehensively addressed [14]. To address this knowledge gap, we performed this retrospective cohort study to investigate the predictive value of GWR on brain CT for DEACMP.

## 2. Methods

This retrospective cohort study followed the principles of the Helsinki Declaration and has been approved by the Ethics Committee of Cangzhou Central Hospital (No. 2020-016-02).

### 2.1. Patients

A total of 352 patients with acute CO poisoning who were admitted to the adult emergency department of the Cangzhou Central Hospital from May 2010 to May 2020 were enrolled in this retrospective cohort study. The inclusion criteria were as follows: (1) acute CO poisoning defined as exposure to CO from charcoal burning and (2) the patients visited the hospital and underwent head CT scans within 12 h from the end of exposure to admission. The exclusion criteria were as follows: (1) the patients were <14 years of age; (2) the patients had a previous history of central nervous system diseases or congenital abnormalities; (3) the condition occurred in combination with other types of poisoning, such as sedative or hypnotic drugs, and alcoholism; and (4) brain CT images were technically inadequate for the determination of cerebral density or were unavailable for evaluation.

### 2.2. Data Collection

We collected demographic data, as well as toxicological and clinical features, as follows: age, gender, heart rate, blood pressure, duration of CO exposure, time from end of exposure to admission, time from end of exposure to brain CT, hyperbaric oxygen therapy, number of hyperbaric oxygen therapy, alanine aminotransferase, creatinine, pH, PO_2_, base excess, COHB, GWR-basal ganglia, GWR-cortices, and GWR-average. The participants were divided into the DEACMP group and the non-DEACMP group in accordance to whether the patients developed DEACMP during 6 months of clinical follow-up [15]. DEACMP was defined as any neurological symptom that newly developed within 6 months after CO exposure; these symptoms could include motor deficits, cognitive decline, dysphagia, dysarthria, dyspraxia, parkinsonism, seizures, psychosis, and mood disorders [16, 17]. Detection of neurological symptom depended on subjective report by patients or their family members. These reports were subsequently confirmed by the medical institution. Regular examination using a standardized cognitive test was not routinely utilized [18].

### 2.3. GWR Determination

**Figure 1:**
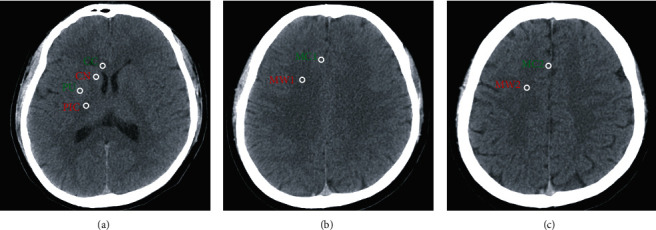
Circular regions of interest were placed bilaterally on brain computed tomography. The regions of interest used for computing the estimation of gray matter to white matter ratio: (a) basal ganglia, (b) centrum semiovale, and (c) high convexity. CC: corpus callosum; CN: caudate nucleus; PU: putamen; PIC: posterior limb of the internal capsule; MC1: medial cortex at centrum semiovale level; MC2: medial cortex at high-convexity area; MW1: medial white matter at centrum semiovale level; MW2: medial white matter at high-convexity area.

A 64-channel scanner (LightSpeed VCT; GE Healthcare, Milwaukee, Wisconsin, USA) was used for all of the CT studies with a 5 mm slice width. Two investigators who were specially trained by a radiologist and were blinded to the clinical outcome measured gray matter and white matter densities in Hounsfield units. As shown in previous studies, in this study, the observers identified comparable brain slices at three levels, including the basal ganglia, centrum semiovale, and high convexity (Figure 1). Furthermore, regions of interest that consisted of circular areas (0.1 cm^2^) were placed bilaterally in the caudate nucleus (CN), putamen (PU), posterior limb of the internal capsule (PIC), forceps minor of the corpus callosum (CC), medial cortex (MC1), and medial WM at the level of the MC2 and high-convexity area. The average of six measurements in each site was defined as the Hounsfield units of the site. The GWRs used in previous studies were calculated as follows: GWR − basal ganglia = (CN + PU)/(PIC + CC); GWR − cerebrum = (MC1 + MC2)/(MW1 + MW2); and GWR − average = (GWR1 + GWR2)/2 [8, 19].

### 2.4. Statistical Analysis

Statistical analysis was performed by using SPSS 13.0. Continuous variables were expressed as means and standard deviations or medians (interquartile ranges). Normally distributed data were compared by using the *t*-test, whereas skewed distributed data were compared by using the Mann–Whitney *U* test. Categorical variables were presented as percentages and compared via the Chi-squared test or Fisher's exact test. Pearson's correlation coefficients were computed for correlation analysis. Univariate and multivariate logistical regression analyses were performed to identify the factors that could be considered as the independent predictors of the development of DEACMP by using the forward stepwise method with the likelihood ratio test. Receiver operating characteristic curves were constructed to establish the cut-off points of variables for optimal sensitivity and specificity in predicting DEACMP development. *P* values of less than 0.05 indicated statistical significance.

## 3. Results

### 3.1. Patient Characteristics

In this study, a total of 352 patients with acute CO poisoning were identified. A total of 16 and 336 patients were allocated to the DEACMP and non-DEACMP groups, respectively. The baseline characteristics of the 352 patients are summarized in Table 1. The GWR-basal ganglia, GWR-average, and GWR-cerebrum in the DEACMP group were lower than that in the non-DEACMP group. Patients with DEACMP had longer duration of CO exposure, faster respiration rate, higher number of hyperbaric oxygen therapy sessions, and worse base excess compared with patients without DEACMP. No significant differences in age, gender, heart rate, mean arterial pressure, time from end of exposure to admission, time from end of exposure to brain CT, hyperbaric oxygen therapy, alanine aminotransferase, creatinine, pH, PO_2_, base excess, and COHB were observed between the DEACMP and non-DEACMP groups.

### 3.2. Correlation Analysis and Logistic Regression Analyses

Correlation analysis indicated that GWR-basal ganglia (*r* = 0.276; *P* < 0.001), GWR-average (*r* = 0.200; *P* < 0.001), and GWR-cerebrum (*r* = 0.163; *P* = 0.002) were correlated with DEACMP. Multivariable logistic regression analysis showed that GWR-basal ganglia (*P* < 0.001), GWR-cortices (*P* = 0.008), and GWR-average (*P* = 0.001) were independent risk factors of DEACMP (Table 2).

### 3.3. ROC Curve Analysis for DEACMP

**Figure 2:**
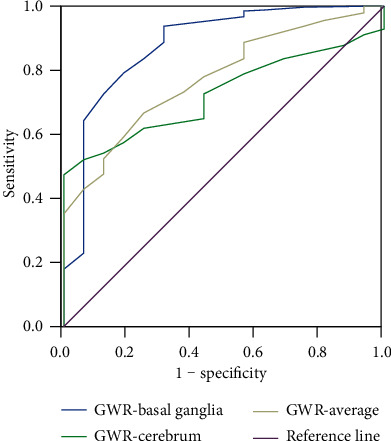
Area under the receiver operating characteristic curve analysis. GWR: gray-matter–white-matter ratio.

Compared with GWR-cerebrum and GWR-average, GWR-basal ganglia had a higher area under the curve of 0.881 (95% CI: 0.783–0.983), thereby suggesting that GWR-basal ganglia had a moderate predictive value for DEACMP. The cut-off value of GWR-basal ganglia was 1.055, and the sensitivity and specificity values were 93.8% and 68.7%, respectively (Figure 2; Table 3).

## 4. Discussion

The morbidity, mortality, and disability rates of DEACMP remain high given the lack of its diagnostic criteria and effective treatment. Therefore, a major challenge for physicians is to identify patients who are likely to develop DEACMP and to prevent DEACMP in patients with acute CO poisoning. This study was the first to demonstrate that GWR could serve as an indicator for predicting DEACMP. Among GWR-basal ganglia, GWR-average, and GWR-cerebrum, GWR-basal ganglia was the best predictor of DEACMP with high sensitivity (93.8%) and high specificity (68.7%).

The worldwide epidemiology of CO poisoning revealed that the incidence of DEACMP is in the range of 3%–40% [6]. In a study involving 281 patients with CO poisoning, Demirtaș et al. [20] reported that 7% developed DEACMP. Cha et al. [21] evaluated the morbidity of DEACMP in 98 patients with CO poisoning and found that the incidence of DEACMP was 8.2%. Kim et al. [22] reported that among 102 patients with CO poisoning, 9.8% had DEACMP. The incidence of DEACMP in the present study was 4.5% (16/352), and this finding was consistent with that in previous reports.

Du et al. [15] measured CT value within a region of interest (2–5 cm^2^) in the following brain regions: bilateral centrum semiovale; white matter of the frontal, parietal, occipital and temporal lobes; basal ganglia; thalamus; capsula interna; cerebral peduncle; pons; and cerebellum. The area under the ROC curve was 0.700, with a 95% confidence interval of 0.584–0.817. Gray matter, with its higher initial water content, was more strongly affected than white matter, because even small changes in water content have a much more pronounced effect on X-ray attenuation [23]. Therefore, GWR would be a more sensitive indicator of the loss of distinction than measurement of either alone [24]. GWR-cerebrum, GWR-average, and GWR-basal ganglia are valuable for the prediction of DEACMP. Among these, GWR-basal ganglia was the best predictor. The combination of CO with hemoglobin to form COHB, a molecule that is incapable of carrying oxygen to tissue sites, results in tissue hypoxia. The reduction in oxygen-carrying capacity due to elevated COHB levels is exacerbated by impaired perfusion, which results from hypoxic cardiac dysfunctions, and triggers ischemia [25]. The brain, as the main energy-consuming organ [26], is highly sensitive to hypoxia and ischemia. Neuronal death and demyelination are common abnormalities found in patients with CO poisoning [27]. Furthermore, bilateral low-density areas in white matter and gray matter on brain CT coincide with pathological findings, such as softening and degeneration, at autopsy [28]. Additionally, as a result of increased metabolic rate, blood flow, and susceptibility to excitotoxicity, the vulnerability of the basal ganglia to ischemic/hypoxic events is greater than that of other areas in the brain [29]. The higher predictive value of GWR-basal ganglia than that of GWR-average and GWR-cerebrum could be ascribed to these phenomena.

Many previous studies have explored the predictive value of magnetic resonance imaging (MRI) in DEACMP. Otubo et al. [30] indicated that MRI could be used to predict DEACMP. Moon et al. [31] found that abnormal diffusion-weighted MRI is an independent factor for poor long-term neurologic outcomes in acute CO poisoning. Furthermore, Kim et al. [22] showed that diffusion-weighted imaging is an early predictor of DEACMP in acute CO poisoning with sensitivity and specificity of 70% and 80.4%, respectively [22]. The present study revealed that GWR-basal ganglia could be used to predict DEACMP with high sensitivity (93.8%) and high specificity (68.7%). However, the predictive values of GWR on CT and MRI require further comparison.

This study has several limitations. First, this study was a single-center study, and its sample size was small. Therefore, the generalizability of our findings to other patient populations is limited. Second, this study was a retrospective study. Thus, some data, such as GCS score, were not evaluated. A well-designed prospective study is necessary to compensate for these limitations.

In conclusion, we determined that GWR, especially GWR-basal ganglia, could serve as an indicator for the prediction of DEACMP. Further research is needed to validate our results.

## Figures and Tables

**Table 1:** General characteristics upon arrival of the study group.

	DEACMP group (*n* = 16)	Non-DEACMP group (*n* = 336)	*P*
Age (years)	60.50 (15.00)	59.00 (22.00)	0.343
Gender (male/female)	8/8	146/190	0.606
Heart rate (beats/min)	96.50 (22)	86.00 (20.00)	0.060
Respiration rate (breaths/min)	20.00 (3.00)	18.50 (2.00)	0.003
Mean arterial pressure (mmHg)	98.50 (14.00)	97.00 (19.00)	0.407
Duration of CO exposure (h)	8.84 (2.13)	4.00 (5.25)	<0.001
Time from end of exposure to admission (h)	3.77 (2.20)	3.51 (1.90)	0.363
Time from end of exposure to brain CT (h)	3.00 (1.98)	2.46 (1.45)	0.092
Hyperbaric oxygen therapy (yes/no)	14/2	267/69	0.434
Number of hyperbaric oxygen therapy sessions	10 (12)	6 (8)	0.038
Alanine aminotransferase (U/L)	20.80 (31.43)	16.10 (11.15)	0.082
Creatinine (*μ*mol/L)	67.00 (29.75)	62.00 (23.00)	0.225
pH	7.40 (0.06)	7.40 (0.05)	0.611
PO_2_ (mmHg)	93.85 (54.83)	88.45 (40.03)	0.667
Base excess (mmol/L)	-4.45 (4.58)	-1.55 (3.40)	0.002
COHB (%)	29.35 (10.45)	28.20 (10.80)	0.721
GWR-basal ganglia	1.05 (0.07)	1.12 (0.06)	<0.001
GWR-cortices	1.04 (0.06)	1.11 (0.13)	0.002
GWR-average	1.06 (0.06)	1.10 (0.08)	<0.001

DEACMP: delayed encephalopathy after acute carbon monoxide poisoning; GWR: gray-matter–white-matter ratio; CO: carbon monoxide; COHB: carboxyhemoglobin. Data are expressed as the mean ± standard deviation, median (interquartile range), or number of patients (percentage).

**Table 2:** Logistic regression analysis for DEACMP.

	*β*	Std. error	Wald	*P* value	Exp (*β*)	95% CI
*Univariate analysis*						
Age	0.019	0.017	1.274	0.259	1.019	0.986–1.053
Gender	0.263	0.512	0.265	0.607	1.301	0.477–3.550
Heart rate	0.032	0.018	3.322	0.068	1.033	0.998–1.069
Respiration rate	0.006	0.012	0.252	0.615	1.006	0.983–1.030
Mean arterial pressure	0.000	0.008	0.001	0.971	1.000	0.984–1.017
Exposure time	0.207	0.059	12.108	0.001	1.230	1.094–1.382
Time from end of exposure to admission	0.072	0.166	0.186	0.667	1.074	0.776–1.488
Time from end of exposure to brain CT	-0.008	0.042	0.039	0.843	0.992	0.914–1.076
Hyperbaric oxygen therapy	0.593	0.786	0.596	0.440	1.089	0.402–8.148
Number of hyperbaric oxygen therapy sessions	0.074	0.030	6.105	0.013	1.077	1.015–1.142
Alanine aminotransferase	0.043	0.015	7.696	0.006	1.044	1.013–1.076
Creatinine	0.012	0.010	1.430	0.232	1.012	0.993–1.031
pH	-1.305	4.753	0.075	0.784	0.271	0.000–3016.406
PO_2_	0.001	0.005	0.056	0.813	1.001	0.991–1.012
Base excess	-0.145	0.062	5.381	0.020	0.865	0.766–0.978
COHB (%)	-0.022	0.030	0.523	0.470	0.978	0.922–1.038
GWR-basal ganglia	-43.274	8.695	24.771	<0.001	0.000	0.000–0.000
GWR-cortices	-10.989	4.103	7.174	0.007	0.000	0.000–0.052
GWR-average	-21.130	6.327	0.001	<0.001	0.000	0.000–0.000
*Multivariate analysis (model 1)*						
Exposure time	0.286	0.084	11.653	0.001	1.330	1.129–1.567
Number of hyperbaric oxygen therapy sessions	0.077	0.048	2.508	0.113	1.080	0.982–1.187
Alanine aminotransferase	0.053	0.022	5.615	0.018	1.054	1.009–1.102
GWR-basal ganglia	-50.272	10.602	22.484	<0.001	0.000	0.000–0.000
*Multivariate analysis (model 2)*						
Exposure time	0.218	0.068	10.275	0.001	1.243	1.088–1.420
Number of hyperbaric oxygen therapy sessions	0.089	0.036	5.928	0.015	1.093	1.017–1.174
Alanine aminotransferase	0.044	0.018	5.747	0.017	1.045	1.008–1.084
GWR-cortices	-10.967	4.109	7.122	0.008	0.000	0.000–0.054
*Multivariate analysis (model 3)*						
Exposure time	0.232	0.074	9.841	0.002	1.261	1.091–1.457
Number of hyperbaric oxygen therapy sessions	0.094	0.040	5.391	0.020	1.098	1.015–1.189
Alanine aminotransferase	0.052	0.020	6.553	0.010	1.053	1.012–1.096
GWR-average	-24.212	7.351	10.847	0.001	0.000	0.000–0.000

CI: confidence interval; DEACMP: delayed encephalopathy after acute carbon monoxide poisoning; GWR: gray-matter–white-matter ratio; COHB: carboxyhemoglobin.

**Table 3:** Receiver operating characteristic curve analysis.

Variable	Area under ROC curve	95% confidence interval	Cut-off	Sensitivity (%)	Specificity (%)	Youden index
GWR-basal ganglia	0.881	0.783–0.983	1.055	93.8	68.7	0.625
GWR-average	0.776	0.681–0.871	1.085	66.7	75.0	0.417
GWR-cerebrum	0.726	0.649–0.802	1.115	52.7	100	0.473

GWR: gray-matter–white-matter ratio; ROC: receiver operating characteristic.

## Data Availability

The datasets used and/or analyzed during the current study are available from the corresponding author on reasonable request.
